# Prognostic significance of NTMT1 and its association with tumor progression in oral squamous cell carcinoma

**DOI:** 10.3389/fonc.2026.1730415

**Published:** 2026-03-11

**Authors:** Lijuan Li, Aixiu Gong, Lingling Xiao, Genxiong Tang

**Affiliations:** 1 Department of Stomatology, Children’s Hospital of Nanjing Medical University, Nanjing, China; 2 Department of Stomatology, Nanjing Drum Tower Hospital, Affiliated Hospital of Medical School, Nanjing University, Nanjing, China

**Keywords:** immune cell, m6A methylation, NTMT1, oral squamous cell carcinoma, prognosis

## Abstract

**Background:**

Oral squamous cell carcinoma (OSCC) is a common malignant tumor of the oral cavity that significantly impacts patients’ quality of life and presents substantial economic challenges due to its high incidence and mortality.

**Methods:**

Weighted gene co-expression network analysis and differential expression gene screening were applied to identify key genes involved in OSCC progression. The expression of NTMT1 in OSCC was examined using The Cancer Genome Atlas database and the Human Protein Atlas database. One-way analysis of variance was used to compare NTMT1 expression among patients with different pathological statuses. Kaplan-Meier survival analysis and Cox regression analysis were performed to investigate the relationship between NTMT1 and prognosis. Gene Ontology analysis and gene set enrichment analysis (GSEA) were conducted to explore the functions and pathways of NTMT1. The correlations between NTMT1 expression and m6A-related genes as well as immune cells were analyzed using R software. Additionally, *in vitro* experiments were carried out to verify the effect of NTMT1 overexpression on the proliferation, migration, and invasion of OSCC cells.

**Results:**

NTMT1 was found to be upregulated in OSCC tissues, with higher expression correlating with poorer survival outcomes. Statistical analyses demonstrated significant associations between NTMT1 expression and various clinicopathological characteristics, including pathologic stage and lymphovascular invasion. Univariate Cox regression analysis indicated NTMT1 as a significant risk factor for OSCC, although multivariate analysis did not confirm its independent prognostic value. Functional analysis revealed NTMT1-associated genes involved in lipid transport, cell adhesion, and DNA repair. GSEA highlighted pathways such as cell cycle checkpoints and Notch signaling. Additionally, NTMT1 expression was positively correlated with m6A methylation-related genes and specific immune cell infiltrations. NTMT1 overexpression promoted the proliferation, migration and invasion of OSCC cells.

**Conclusion:**

NTMT1 might play a crucial role in OSCC progression and have the potential to serve as a biomarker for prognosis and therapeutic targeting.

## 1 Introduction

Oral squamous cell carcinoma (OSCC) represents the most prevalent form of malignancy within the oral cavity, accounting for over 90% of oral cancers and contributing substantially to global morbidity and mortality (1, 2). This neoplasm typically arises from the mucosal linings of the oral cavity, including the tongue, floor of mouth, buccal mucosa, and oropharyngeal regions (3). Despite advances in public health awareness and early detection strategies, the five-year survival rate for OSCC remains dismally low, especially in advanced stages, where it can decline to approximately 50% (1). Early-stage lesions are often asymptomatic or non-specific, resulting in delayed diagnosis and suboptimal outcomes (4). Existing biomarkers and imaging techniques have not achieved sufficient sensitivity and specificity for early detection or accurate prognostication (5). Therefore, there is a compelling need to characterize additional molecules with robust prognostic value and mechanistic relevance to tumor biology.

NTMT1 (N-terminal methyltransferase 1) is an enzyme that catalyzes the N-terminal α-methylation (Nα-methylation). It recognizes the specific X-P-K/R motif and transfers a methyl group from S-adenosylmethionine to the N-terminus of substrate proteins (6). This modification affects protein stability, protein-protein interactions, and protein-DNA interactions, thereby regulating processes such as cell proliferation, immune response, and DNA damage repair. Meng et al. (7) verified the Nα-methylation effect of NTMT1 on protein arginine deiminase 1 (PAD1), and found that this modification significantly prolonged the half-life of PAD1 and regulated its protein interactions, but had no significant impact on its enzymatic activity or cellular localization. In addition, NTMT1-mediated Nα-methylation affects the functions of chromosome condensation regulators and the oncoprotein SET, suggesting its potential regulatory role in the cell cycle and tumorigenesis (8). In colorectal cancer, the cell-free DNA in plasma test (MethyDT) based on the methylation of NTMT1 and MAP3K14-AS1 shows high sensitivity and specificity, and can be used for non-invasive diagnosis (9). NTMT1 was confirmed to be positively correlated with the progression of cervical cancer and could significantly promote proliferation and migration of tumor cells (10). However, there is little known about the role of NTMT1 in OSCC progression.

This research aims to clarify the clinical relevance and underlying biological functions of NTMT1 in OSCC, thereby advancing our understanding of its potential as a prognostic biomarker and informing new avenues for targeted therapy. The integration of bioinformatics methodologies and cell experiments not only enhances the robustness of the findings but also lays the groundwork for future translational studies about NTMT1. Through this systematic analysis, the study aspires to contribute meaningfully to the ongoing efforts to improve prognostic evaluation and therapeutic interventions for OSCC.

## 2 Materials and methods

### 2.1 Data acquisition

The GSE37991 dataset was downloaded from the GEO database (https://www.ncbi.nlm.nih.gov/geo/query/acc.cgi), which includes 40 OSCC tissue samples and 40 adjacent non-tumor tissue samples. RNA-seq data processed by the STAR pipeline, along with corresponding clinical data, were downloaded and organized from the TCGA-HNSC project in the TCGA database (https://portal.gdc.cancer.gov). TPM-formatted expression data were extracted from the RNA-seq dataset. Samples without matching clinical information were excluded. From the remaining samples, those derived from oral cancer sites (alveolar ridge, base of tongue, buccal mucosa, floor of mouth, hard Palate, oral cavity, oral tongue) were retained, while samples from non-oral cancer sites (hypopharynx, larynx, lip, oropharynx, tonsil) were removed. The resulting cohort was defined as the TCGA-OSCC cohort.

### 2.2 Weighted gene co-expression network analysis

Based on the GES37991 gene expression matrix, genes with low expression (e.g., FPKM < 1 in 80% of samples) and low variation (CV < 0.15) were first filtered out. After normalization, the remaining genes were used for subsequent analysis. Using the R “WGCNA” package, the optimal soft-thresholding power β was selected to satisfy the scale-free network property, and a weighted adjacency matrix was constructed. Gene modules were obtained via hierarchical clustering based on TOM dissimilarity followed by dynamic tree cutting, with modules showing ME correlation coefficient > 0.75 merged. The correlation between module eigengenes (ME) and phenotypes was calculated to screen significantly associated modules. Core genes were selected based on the criteria of MM > 0.8 and GS > 0.2.

### 2.3 Differentially expressed genes between NTMT1 high-expression and low-expression groups

Expression data of NTMT1 were extracted from the GSE37991/TCGA-OSCC and stratified into high- and low-expression groups based on the median expression value of NTMT1. Differential expression analysis was performed on the raw counts matrix using the DESeq2 package following standard protocols. Additionally, the raw counts matrix was normalized using the Variance Stabilizing Transformation (VST) method provided by the DESeq2 package.

### 2.4 NTMT1 expression in pan-cancer and OSCC

RNA-seq data processed by the STAR pipeline for 33 tumor projects were downloaded from the TCGA database, and TPM-formatted data were extracted. Based on the characteristics of the data format, appropriate statistical methods (Wilcoxon rank sum test, unpaired t-test, paired t-test) from the stats and car packages were selected to analyze the expression of NTMT1 mRNA in pan-cancer and OSCC. The Human Protein Atlas (HPA) database provides immunohistochemical results showing the expression of NTMT1 protein in OSCC and normal oral tissues.

### 2.5 Study on the correlation between NTMT1 and clinical characteristics

In this study, statistical calculations were conducted with the “stats” package, hypothesis testing was performed via the “car” package, and data visualization was implemented using the “ggplot2” package. The significance of all statistical results was determined through one-way ANOVA.

### 2.6 Survival analysis of NTMT1

In the TCGA-OSCC cohort, patients were divided into high-expression and low-expression groups using the median expression level of NTMT1 as the threshold. The R “survival” package was employed to perform the proportional hazards assumption test and fit the survival regression model. Finally, the “survminer” package combined with the “ggplot2” package was used to visualize the results of the survival analysis.

### 2.7 Cox regression analysis

The survival package was used to perform proportional hazards assumption tests and Cox regression analyses on the pathological features of data from the TCGA-OSCC cohort. In the univariate Cox regression analysis, samples meeting the predefined threshold of *P* < 0.05were included in the multivariate Cox regression analysis for model construction.

### 2.8 Gene ontology analysis

Genes related to NTMT1 were subjected to ID conversion using the “org.Hs.eg.db” package, followed by enrichment analysis with the “clusterProfiler” package. The results of the enrichment analysis were visualized using the ggplot2 package.

### 2.9 Gene set enrichment analysis

DEGs between the NTMT1 high-expression and low-expression groups from TCGA-OSCC cohort were used as the analysis objects. Reference gene sets, including Hallmark, KEGG, and GO-BP gene sets from the MsigDB database, were selected for the analysis. The core parameters were set as follows: 1000 permutations were performed to reduce false positives; the gene set size threshold was set to 15-500 (to exclude excessively small or large gene sets and ensure the reliability of enrichment results); and the significance level was defined as adjusted P-value < 0.05. During the analysis, the enrichment score (ES) was calculated to quantify the enrichment degree of target gene sets in the ranked list of DEGs, and permutation tests were used to determine the statistical significance of the enrichment results.

### 2.10 Association between NTMT1 expression and m6A methylation in OSCC

Tissue samples in TCGA-OSCC cohort were divided into NTMT1 high-expression and low-expression groups. The expressions of m6A related genes in each group were compared through T test. The correlations between NTMT1 and m6A related genes were analyzed by Spearman method.

### 2.11 Connection of NTMT1 expression to infiltrating immune cells in OSCC

Based on the ssGSEA algorithm implemented in the R package “GSVA”, we calculated the correlation between NTMT1 and immune infiltration in TCGA-OSCC using markers for 24 immune cell types derived from a study published in Immunity (11). Samples were stratified by NTMT1 expression levels, and the enrichment scores of each immune cell type were compared between the two groups. Furthermore, immune infiltration profiles in TCGA-OSCC were evaluated using the core CIBERSORT algorithm with markers for 22 immune cell types obtained from the CIBERSORTx website (https://cibersortx.stanford.edu/).

### 2.12 Experimental methods

These methods are detailed in the **Supplementary Methods** section.

## 3 Results

### 3.1 Screening of key genes in the progress of OSCC

**Figure 1:**
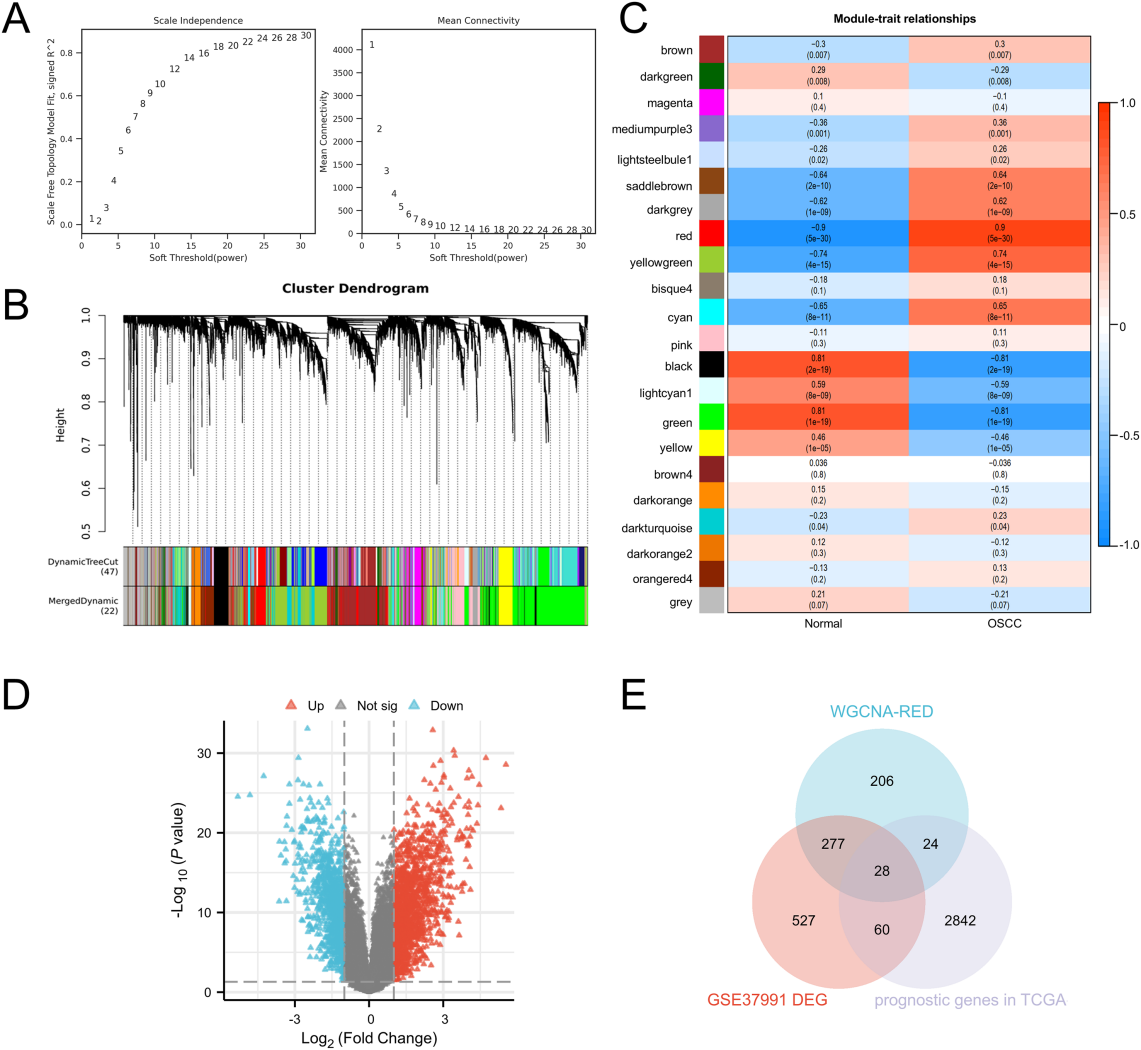
Screening for key genes in OSCC progression. **(A)** Parameters for WGCNA network construction. **(B)** Gene cluster trees constructed from the TOM matrix constructed from the weighted correlation coefficients. **(C)** Heat map of trait module association. **(D)** Volcano map of differential expressed genes of GSE37991. **(E)** Venn diagram of key genes in OSCC.

We constructed a WGCNA using the “WGCNA” package in R software to perform sample clustering for GSE37991. A soft-thresholding power of β=18 was selected to ensure the scale-free topology of the network (**Figure 1A**). A total of 8, 074 genes were ultimately divided into 22 modules (**Figure 1B**). Notably, the Red module exhibited the most significant module significance (cor = -0.92, P < 0.001, **Figure 1C**). Subsequently, we identified differentially expressed genes (DEGs) between normal oral tissues and OSCC tissues in the GSE37991 dataset, resulting in 892 DEGs (**Figure 1D**). We then screened for prognosis-related genes in the TCGA-OSCC cohort, yielding 2, 954 prognosis-associated genes (**Supplementary Table 1**). Finally, a Venn diagram was used to identify the overlapping genes among the Red module genes from WGCNA, the identified DEGs, and the prognosis-related genes, leading to the discovery of 28 common genes, namely PLAU, NEK6, TRIML2, ARHGEF39, MED15, GPM6B, HOXA1, RCE1, CELSR3, STC2, PDIA5, ZIC2, HOXB7, DSG2, CA9, CCL11, RAG1, BIK, ZIC5, ITGA5, TK1, TINAGL1, C11orf87, SCG5, RTP3, CCNA1, NTMT1, GDF15 (**Figure 1E**). Through a comprehensive literature review, we identified NTMT1 as the target for further investigation.

### 3.2 Upregulated NTMT1 in OSCC tissues and its prognostic value

**Figure 2:**
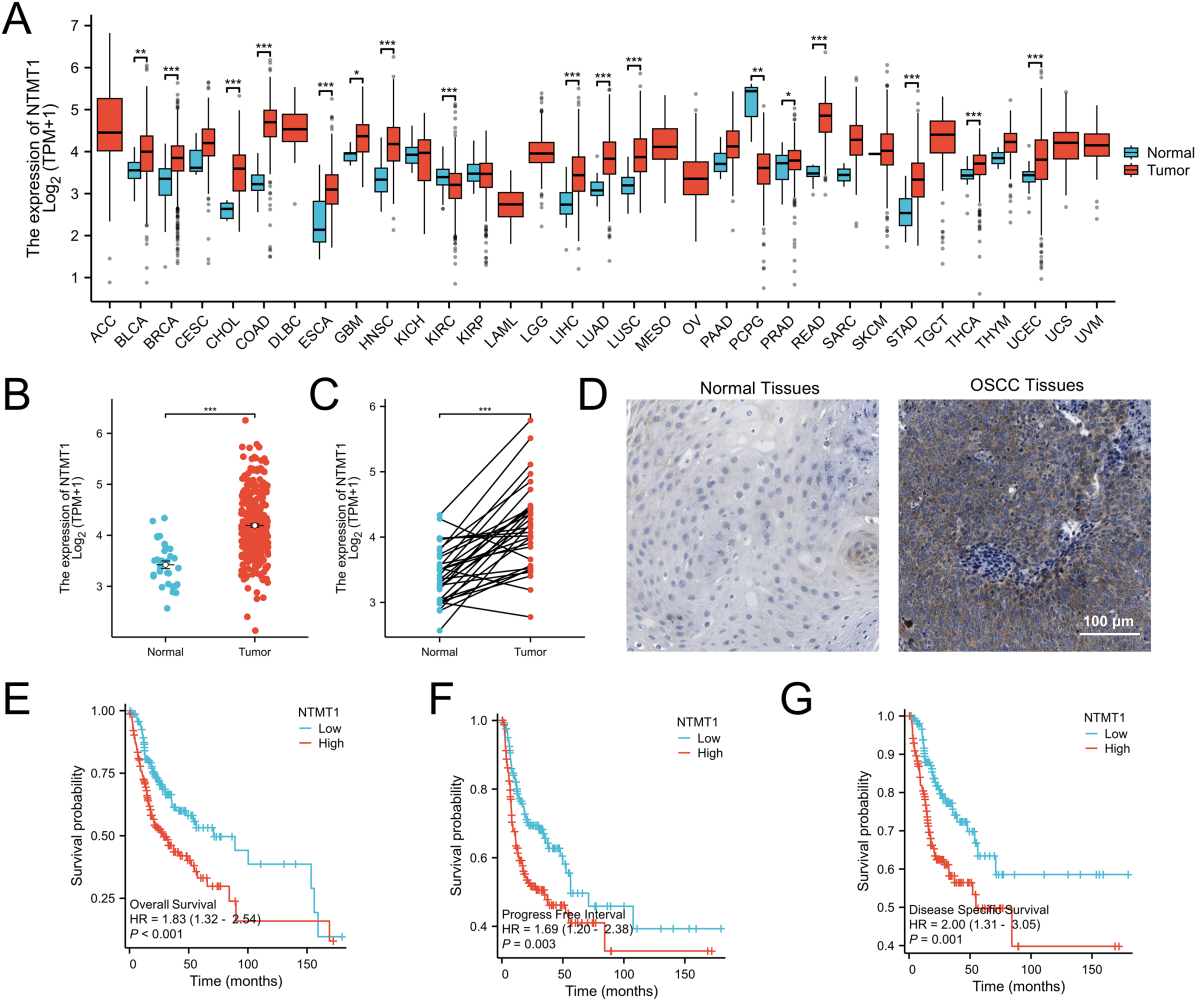
Expression of NTMT1 in OSCC. **(A)** The NTMT1 expression in pan-cancer based on TCGA database. **(B, C)** The comparison of NTMT1 levels in normal tissues and OSCC tissues using t test **(B)** and paired t test **(C)**. **(D)** The protein expression of NTMT1 in normal oral tissues and OSCC tissues from the HPA database. **(E–G)** The overall survival **(E)**, progression-free survival **(F)**, and disease-specific survival **(G)** of OSCC patients with different NTMT1 expressions. *P<0.05, **P<0.01, ***P<0.001.

Data from The Cancer Genome Atlas (TCGA) database revealed that NTMT1 is highly expressed in 15 types of tumors, with reduced expression observed only in kidney renal clear cell carcinoma and pheochromocytoma and paraganglioma (**Figure 2A**). In OSCC, both unpaired and paired t-tests confirmed that NTMT1 expression is significantly higher in tumor tissues compared to normal oral tissues (**Figures 2B, C**). Immunohistochemical results from the HPA database demonstrated that NTMT1 protein is localized in the cytoplasm, with higher expression levels in OSCC tissues than in normal oral tissues (**Figure 2D**). Kaplan-Meier survival analysis indicated that patients with high NTMT1 expression had shorter survival times than those with low NTMT1 expression, as evidenced by overall survival, progression-free survival, and disease-specific survival analyses (**Figures 2E–G**).

### 3.3 Associations between NTMT1 expression and clinicopathological characteristics in OSCC patients

**Figure 3:**
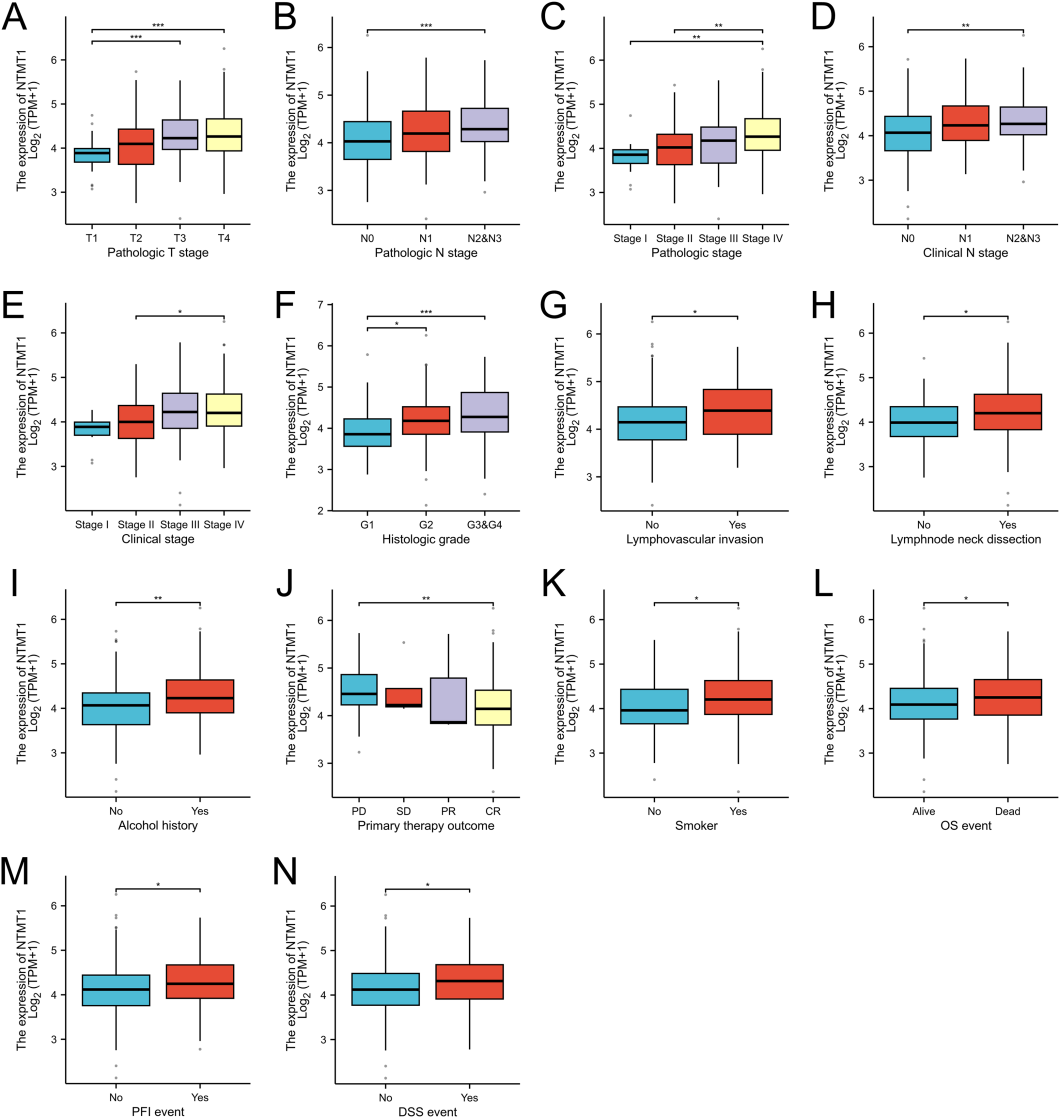
The correlation between NTMT1 expression and clinicopathological characteristics in OSCC patients. **(A)** Pathologic T stage. **(B)** Pathologic N stage. **(C)** Pathologic stage. **(D)** Clinical N stage. **(E)** Clinical stage. **(F)** Histologic grade. **(G)** Lymphovascular invasion. **(H)** Lymph node neck dissection. **(I)** Alcohol history. **(J)** Primary therapy outcome. **(K)** Smoking status. **(L)** OS event. **(M)** PFI event. **(N)** DSS event. *P<0.05, **P<0.01, ***P<0.001.

**Figure 4:**
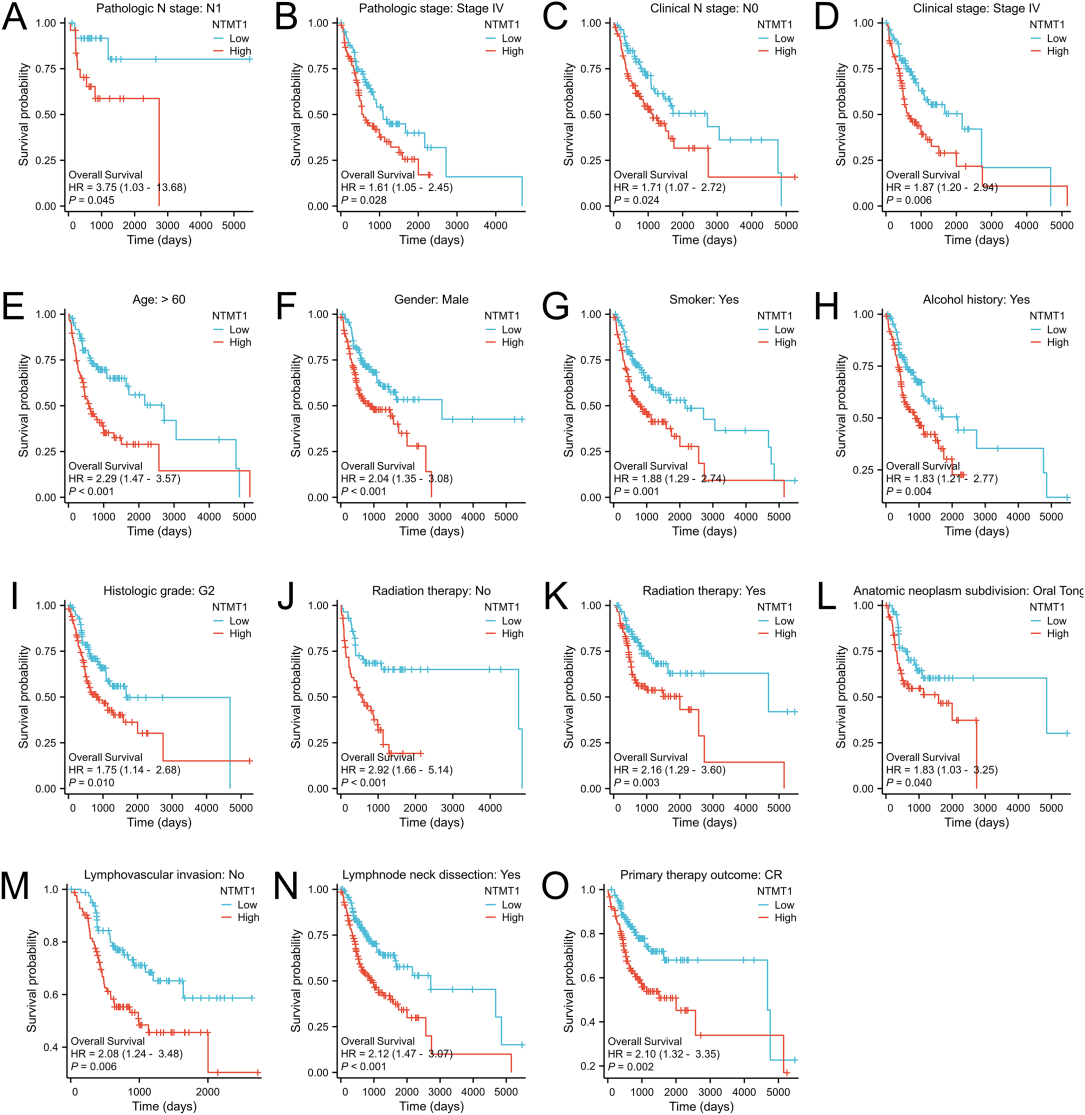
Prognostic subgroup analyses of OSCC patients with different clinicopathological status. **(A)** Pathologic N1 stage, **(B)** Pathologic stage IV, **(C)** Clinical N0 stage, **(D)** Clinical stage IV, **(E)** Aged >60 years, **(F)** Female patients, **(G)** Smokers, **(H)** Alcohol drinkers, **(I)** Histologic medium grade (G2), **(J)** No radiation therapy, **(K)** Radiation therapy. **(L)** Tumors located in the oral tongue, **(M)** No lymphovascular invasion, **(N)** With lymph node neck dissection, **(O)** With a complete response (CR).

NTMT1 expressions among patients with different pathological statuses were compared in the TCGA-OSCC cohort and found that NTMT1 expression was associated with pathologic T stage, pathologic N stage, pathologic stage, clinical N stage, clinical stage, histologic grade, lymphovascular invasion, lymph node neck dissection, alcohol history, primary therapy outcome, smoking status, OS event, PFI event, and DSS event (**Figures 3A–N**). Subsequently, we performed prognostic subgroup analyses for OSCC patients with different clinicopathological characteristics. The results revealed that in OSCC patients with pathologic N1 stage, pathologic stage IV, clinical N0 stage, or clinical stage IV, those with high NTMT1 expression had shorter OS than those with low NTMT1 expression (**Figures 4A–D**). In addition, high NTMT1 expression was also indicative of shorter survival in patients aged >60 years, female patients, smokers, alcohol drinkers, those with histologic medium grade (G2), those who received or did not receive radiation therapy, those with tumors located in the oral tongue, those without lymphovascular invasion, those who underwent lymph node neck dissection, and those who achieved a complete response (CR) to primary therapy (**Figures 4E–O**).

### 3.4 Role of NTMT1 in OSCC risk prediction

Univariate Cox regression analysis demonstrated that NTMT1 expression (HR = 1.833, 95% CI: 1.321–2.542, P< 0.001), primary therapy outcome (HR = 0.157, 95% CI: 0.100–0.246, P< 0.001), lymphovascular invasion (HR = 1.711, 95% CI: 1.149–2.548, P = 0.008), radiation therapy (HR = 0.601, 95% CI: 0.421–0.869, P = 0.005), pathologic T3 stage (HR = 3.980, 95% CI: 1.677–9.447, P = 0.002), pathologic T4 stage (HR = 3.717, 95% CI: 1.592–8.676, P = 0.002), pathologic N2/N3 stage (HR = 2.253, 95% CI: 1.523–3.331, P< 0.001), and pathologic stage IV (HR = 2.445, 95% CI: 1.534–3.929, P< 0.001) were significantly associated with the OS of patients with OSCC (**Table 1**). In contrast, multivariate Cox regression analysis confirmed that primary therapy outcome (HR = 0.370, 95% CI: 0.190–0.720, P = 0.003), lymph node neck dissection (HR = 0.138, 95% CI: 0.034–0.554, P = 0.005), and radiation therapy (HR = 0.427, 95% CI: 0.235–0.774, P = 0.005) were independent prognostic factors for OSCC patients. Notably, NTMT1 expression (HR = 1.410, 95% CI: 0.811–2.449, P = 0.223) did not exhibit a significant association with OS in the multivariate model (**Table 1**).

**Table 1:** Univariate and multivariate Cox regression analyses of clinical characteristics in OSCC.

Characteristics	Total (n)	Univariate analysis	Multivariate analysis		
		HR (95% CI)	P value	HR (95% CI)	P value
NTMT1 expression	329				
Low	164	Reference		Reference	
High	165	1.833 (1.321-2.542)	**< 0.001**	1.410 (0.811-2.449)	0.223
Age	329				
≤ 60	156	Reference			
>60	173	1.330 (0.961-1.840)	0.085		
Gender	329				
Female	102	Reference			
Male	227	0.903 (0.645-1.266)	0.555		
Histologic grade	321				
G1	52	Reference			
G2	200	1.453 (0.893-2.364)	0.133		
G3&G4	69	1.681 (0.978-2.891)	0.060		
Alcohol history	321				
No	105	Reference			
Yes	216	1.039 (0.736-1.467)	0.827		
Smoker	323				
No	88	Reference			
Yes	235	1.266 (0.857-1.870)	0.236		
Primary therapy outcome	270				
PD	35	Reference		Reference	
CR	235	0.157 (0.100-0.246)	**< 0.001**	0.370 (0.190-0.720)	**0.003**
Lymphovascular invasion	239				
No	164	Reference		Reference	
Yes	75	1.711 (1.149-2.548)	**0.008**	1.394 (0.786-2.472)	0.256
Lymphnode neck dissection	327				
No	45	Reference		Reference	
Yes	282	0.681 (0.449-1.033)	0.071	0.138 (0.034-0.554)	**0.005**
Radiation therapy	294				
No	115	Reference		Reference	
Yes	179	0.601 (0.421-0.859)	**0.005**	0.427 (0.235-0.774)	**0.005**
Pathologic T stage	304				
T1	29	Reference		Reference	
T2	99	1.756 (0.737-4.185)	0.204	1.272 (0.302-5.355)	0.743
T3	66	3.980 (1.677-9.447)	**0.002**	2.660 (0.579-12.223)	0.209
T4	110	3.717 (1.592-8.676)	**0.002**	2.912 (0.568-14.921)	0.200
Pathologic N stage	275				
N0	117	Reference		Reference	
N1	50	0.747 (0.400-1.394)	0.359	0.494 (0.182-1.343)	0.167
N2&N3	108	2.253 (1.523-3.331)	**< 0.001**	1.233 (0.522-2.914)	0.632
Pathologic stage	298				
I & II	70	Reference		Reference	
III	61	1.513 (0.845-2.709)	0.164	2.711 (0.691-10.641)	0.153
IV	167	2.455 (1.534-3.929)	**< 0.001**	3.178 (0.726-13.908)	0.125

Bold values indicate P < 0.05.

### 3.5 Function analysis of MTMT1 in OSCC

**Figure 5:**
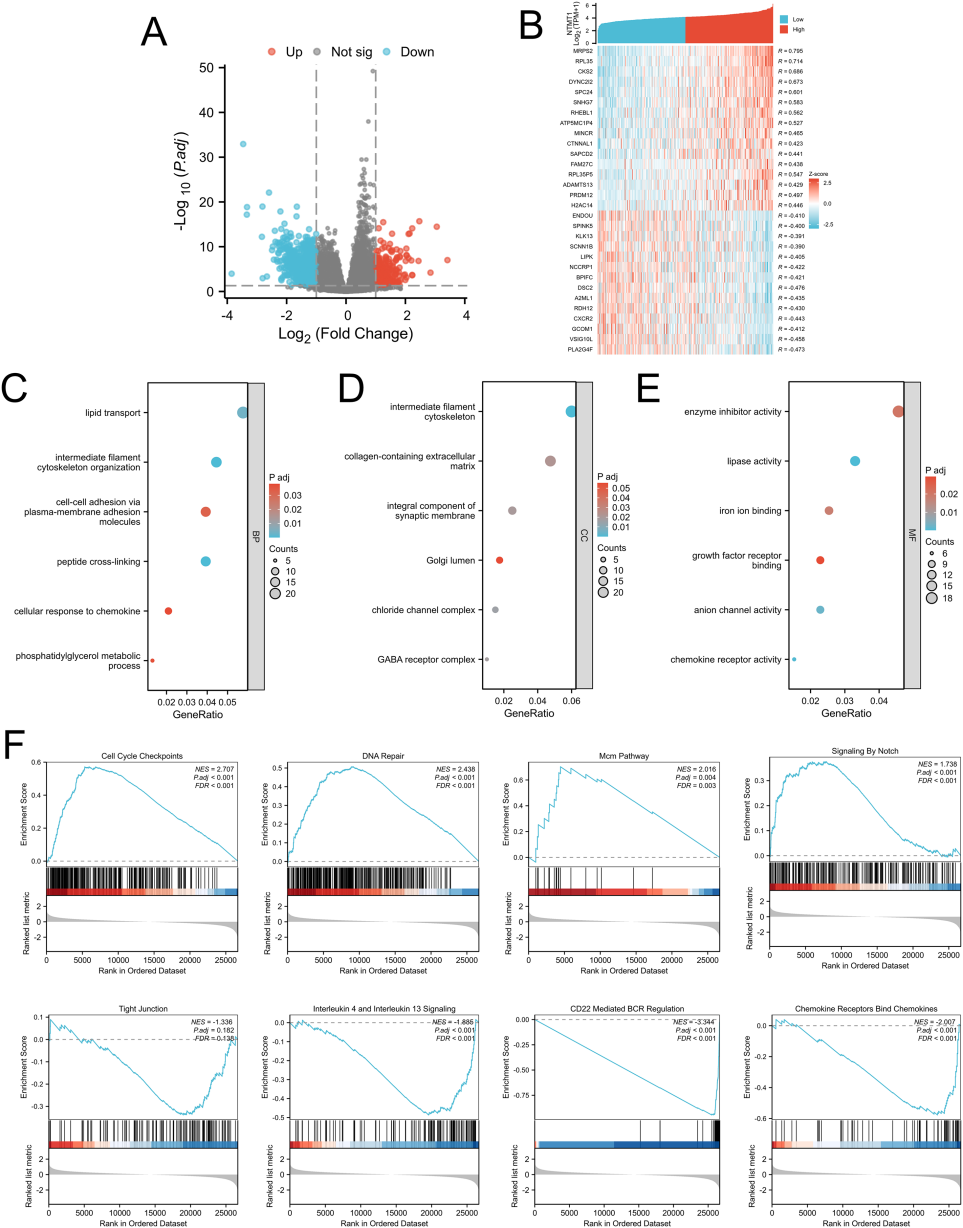
Functional analysis of NTMT1 in OSCC. **(A)** Volcano map of DEGs from TCGA-OSCC. **(B)** Heat map showing the top genes associated withNTMT1 in OSCC. **(C)** BP of NTMT1 related genes. **(D)** CC of NTMT1 related genes. **(E)** MF of NTMT1 related genes. **(F)** GSEA of NTMT1 related genes.

Using the Limma package, we divided the samples in the TCGA-OSCC cohort into high NTMT1 expression group and low NTMT1 expression group based on the median NTMT1 expression level. Subsequent comparison between the two groups identified 1, 083 NTMT1-associated genes (**Figure 5A**), with a subset of these NTMT1-associated genes shown in **Figure 5B**. Gene Ontology (GO) analysis results revealed that the NTMT1-associated genes were involved in 67 Biological Processes (BP), 26 Cellular Components (CC), and 60 Molecular Functions (MF). In the BP category, the NTMT1-associated genes were potentially related to lipid transport, intermediate filament cytoskeleton organization, cell-cell adhesion via plasma-membrane adhesion molecules, and cellular response to chemokine (**Figure 5C**). For the CC category, these genes were enriched in the intermediate filament cytoskeleton, collagen-containing extracellular matrix, chloride channel complex, and GABA receptor complex (**Figure 5D**). Regarding the MF category, NTMT1 might be associated with enzyme inhibitor activity, lipase activity, iron ion binding, and growth factor receptor binding (**Figure 5E**). Furthermore, GSEA results demonstrated that the NTMT1-associated genes were enriched in the upregulated gene sets of Cell cycle checkpoints, DNA repair, MCM pathway, and Signaling by Notch, while being enriched in the downregulated gene sets of Tight junction, Interleukin 4 and Interleukin 13 signaling, CD22 mediated BCR regulation, and Chemokine receptors bind chemokines (**Figure 5F**).

### 3.6 Connection of LDHA expression to m6A modification in EC

**Figure 6:**
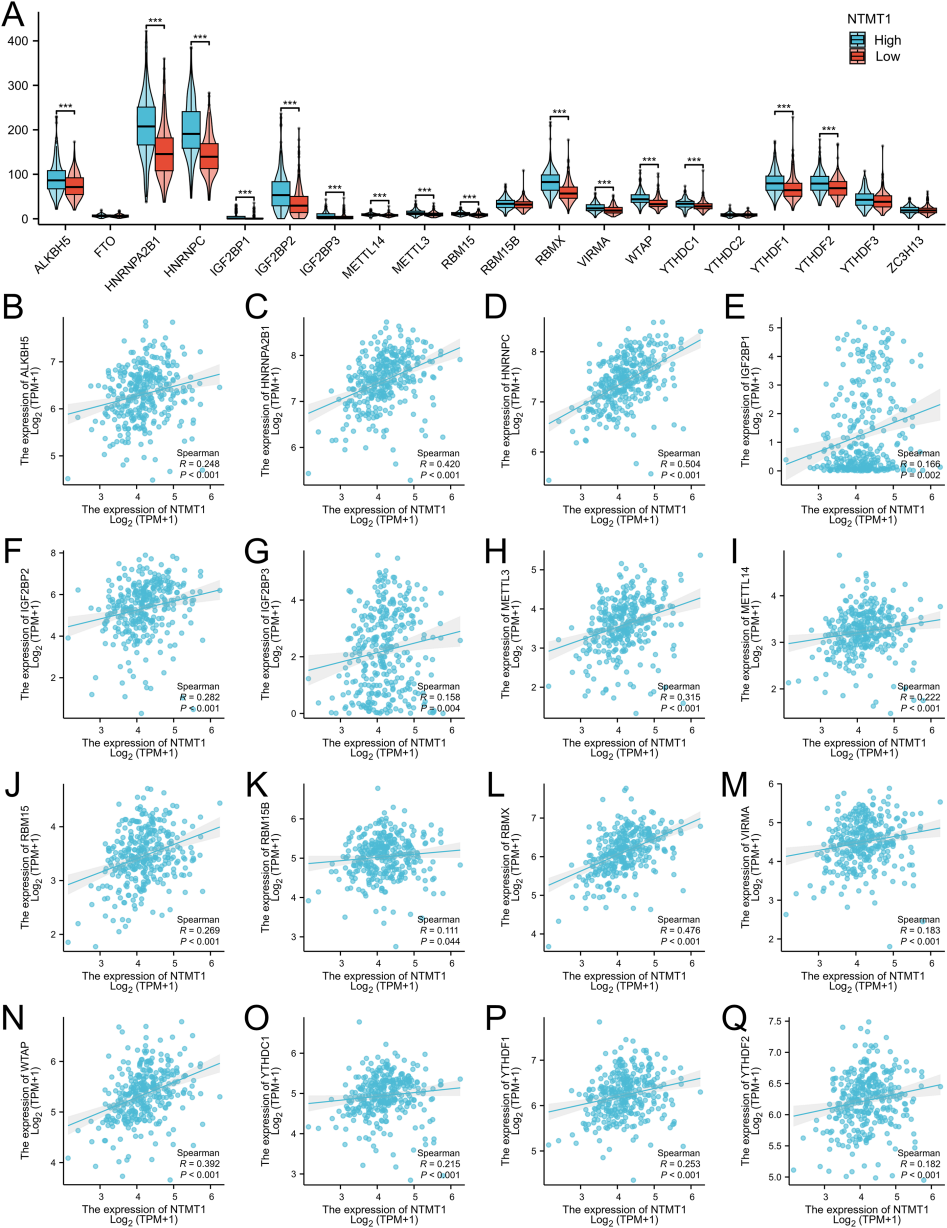
The relationship between NTMT1 expression and m6A modification. **(A)** Differential distribution of m6A related genes in patients with high or low NTMT1 expression. **(B–Q)** Scatter plots were plotted showing the association between NTMT1 and m6Arelated genes ***P<0.001.

In TCGA-OSCC cohort, high NTMT1 expression group had higher ALKBH5, HNRNPA2B1, HNRNPC, IGF2BP1, IGF2BP2, IFG2BP3, METTL14, METTL3, RBM15, RBMX, VIRMA, WTAP, YTHDC1, YTHDF1 and YTHDF2 expressions levels (**Figure 6A**). Spearman correlation analysis showed that NTMT1expression was positively associated with levels of ALKBH5 (R = 0.248, *P* < 0.001), HNRNPA2B1 (R = 0.420, *P* < 0.001), HNRNPC (R = 0.504, *P* < 0.001), IGF2BP1 (R = 0.166, *P* = 0.002), IGF2BP2 (R = 0.282, *P* < 0.001), IFG2BP3 (R = 0.158, *P* = 0.004), METTL14 (R = 0.222, *P* < 0.001), METTL3 (R = 0.315, *P* < 0.001), RBM15 (R = 0.269, *P* < 0.001), RBM15B (R = 0.111, *P* = 0.044), RBMX (R = 0.476, *P* < 0.001), VIRMA (R = 0.183, *P* < 0.001), WTAP (R = 0.392, *P* < 0.001), YTHDC1 (R = 0.215, *P* < 0.001), YTHDF1 (R = 0.253, *P* < 0.001) and YTHDF2 (R = 0.182, *P* < 0.001) (**Figures 6B–Q**).

### 3.7 Relationships between NTMT1 expression and tumor-infiltrating immune cells in OSCC

**Figure 7:**
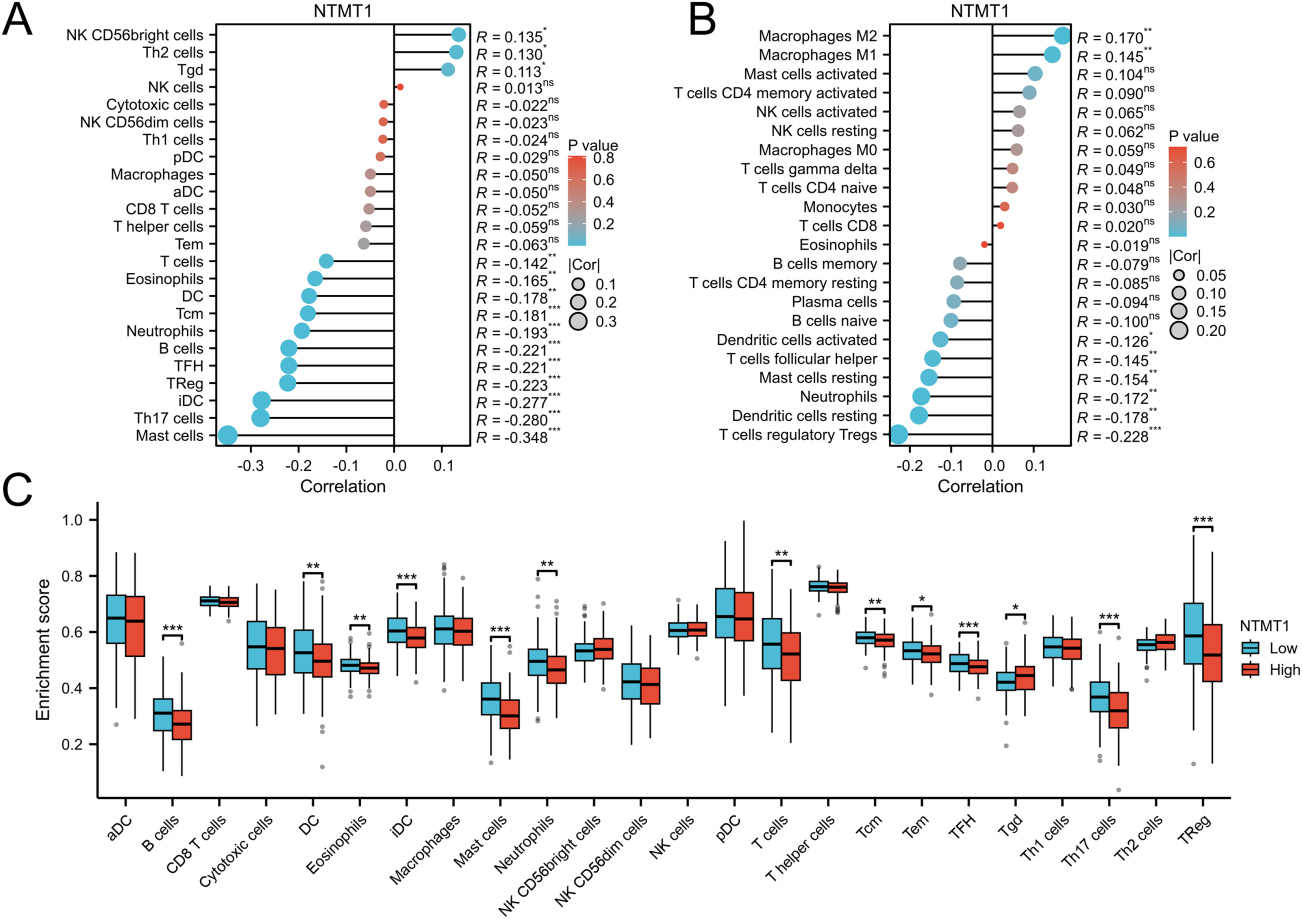
The relationship between NTMT1 expression and immune cell infiltration. **(A, B)** The correlation between NTMT1 and immune cells in OSCC analyzed by the ssGSEA method **(A)** and CIBERSORT algorithm **(B)**. **(C)** Differential distribution of immune cells in patients with different NTMT1 expression. *P<0.05, **P<0.01, ***P<0.001.

Using the ssGSEA method, NTMT1 expression was positively correlated with the infiltration levels of natural killer (NK) CD56bright cells, T helper 2 (Th2) cells, and γδ T cells (Tgd). In contrast, it was negatively correlated with the infiltration of T cells, eosinophils, dendritic cells (DCs), central memory T cells (Tcm), neutrophils, B cells, follicular helper T cells (TFH), regulatory T cells (Tregs), immature DCs (iDCs), Th17 cells, and mast cells (**Figure 7A**). When the CIBERSORT algorithm was applied, NTMT1 expression exhibited a positive association with M2 macrophages and M1 macrophages, while showing a negative association with activated DCs, TFH cells, resting mast cells, neutrophils, resting DCs, and Tregs (**Figure 7B**). Furthermore, group comparison analysis revealed that the high NTMT1 expression group had significantly lower enrichment scores for B cells, DCs, eosinophils, iDCs, mast cells, neutrophils, T cells, Tcm, effector memory T cells (Tem), TFH cells, Th17 cells, and Tregs. Notably, the high NTMT1 group showed a significantly higher enrichment score only for Tgd (**Figure 7C**).

### 3.8 Influence of NTMT1 overexpression on OSCC cells

**Figure 8:**
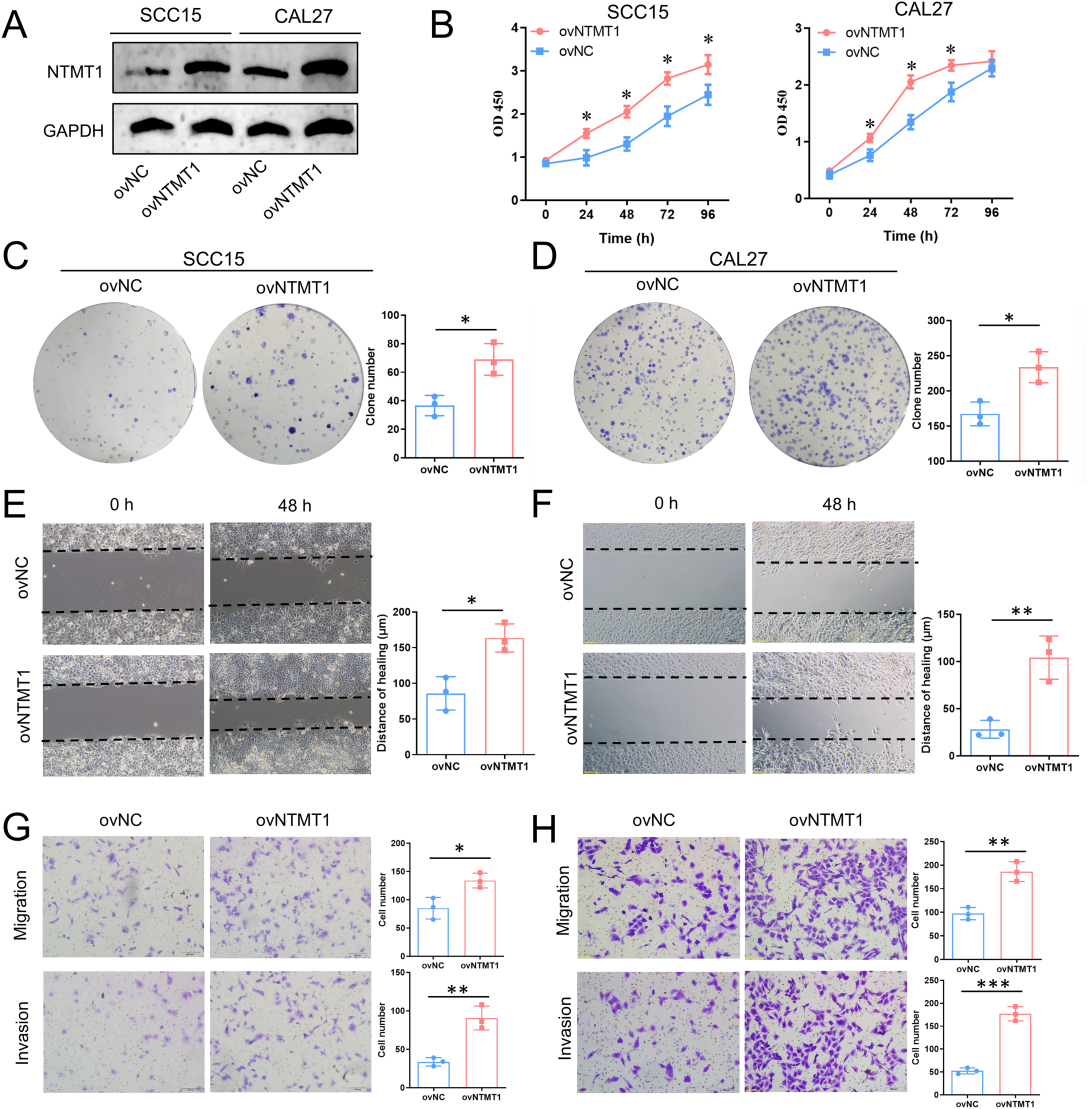
The influence of NTMT1 overexpression on OSCC cell behaviors. **(A)** The upregulated NTMT1 detected by Western Blot. **(B)** The cell proliferation detected by CCK-8 assay. **(C, D)** The cell proliferation detected by clone formation assay. **(E, F)** The cell migration of SCC15 **(E)** and CAL27 **(F)** detected by the wound healing assay. **(G)** The migration (upper panel) and invasion (lower panel) of SCC15 cells detected by the Transwell assay. **(H)** The migration (upper panel) and invasion (lower panel) of CAL27 cells detected by the Transwell assay. *P<0.05, **P<0.01, ***P<0.001.

To further investigate the role of NTMT1 in OSCC, we examined the effect of NTMT1 overexpression on the malignant biological behaviors of OSCC cells. As shown in **Figure 8A**, NTMT1 was successfully overexpressed in the OSCC cell lines SCC15 and CAL27. CCK-8 proliferation assay and colony formation assay demonstrated that NTMT1 overexpression significantly promoted the proliferative capacity of OSCC cells (**Figures 8B–D**). Wound healing assay revealed that NTMT1 overexpression enhanced the planar directional migration and collective migration abilities of OSCC cells (**Figures 8E, F**). Additionally, Transwell migration assay confirmed that NTMT1 overexpression facilitated the three-dimensional independent migration ability of OSCC cells, while Transwell invasion assay showed that NTMT1 overexpression increased the invasive capacity of OSCC cells (**Figures 8G, H**).

## 4 Discussion

OSCC is a prevalent and aggressive malignancy within the head and neck region, characterized by high morbidity and mortality rates. Despite advancements in therapeutic strategies, including surgery, radiation, and chemotherapy, the prognosis for OSCC patients remains poor, primarily due to late-stage diagnoses, treatment resistance, and tumor recurrence (12). The complexity of the tumor microenvironment and the interplay of various genetic and environmental factors contribute to the disease’s aggressive nature (13). Therefore, identifying novel biomarkers and therapeutic targets is crucial for enhancing early detection and improving patient outcomes in OSCC.

Based on a series of bioinformatics analyses and literature reviews, we propose that NTMT1 may play a crucial role in the occurrence and progression of OSCC. NTMT1 is a key methyltransferase that specifically catalyzes the methylation modification at the N-terminus of proteins. This post-translational modification influences protein stability, subcellular localization, and functions such as regulating signal transduction and cell cycle progression. The role of NTMT1 in DNA repair and mitotic regulation suggests that it may be widely involved in the pathological processes of cancer (14, 15). Studies have shown that NTMT1 is highly expressed in cervical cancer (10), retinoblastoma (16), bladder cancer (17), and acute myeloid leukemia (AML) (18). The notable upregulation of NTMT1 in OSCC tissues, as demonstrated in our study, suggests its potential involvement in the tumorigenic processes associated with OSCC.

Moreover, our findings reveal a correlation between NTMT1 expression and adverse clinical characteristics in OSCC patients. This association underscores the relevance of NTMT1 expression in predicting patient outcomes and tailoring treatment strategies. Integrating NTMT1 into clinical decision-making could enable the stratification of patients based on their risk for disease progression and recurrence, thereby optimizing therapeutic approaches (19). Additionally, the use of NTMT1 as a stratifying factor in clinical trials may also facilitate the identification of patient cohorts that are more likely to benefit from targeted therapies, as suggested by prior studies focusing on similar biomarkers in oncology (20). Notably, when NTMT1 expression differences correlate more strongly with survival outcomes in patients with specific pathological features—for example, high NTMT1 expression predicts poor prognosis exclusively in pathologic/clinical stage IV patients—this suggests NTMT1 may participate in critical processes of disease progression, such as promoting invasion and metastasis.

The prognostic value of NTMT1 has been confirmed in various tumors. Lu et al. demonstrated that the upregulation of NTMT1 is associated with a poor prognosis in patients with bladder cancer (17). Li et al. believed that high expression of NTMT1 indicated a poorer survival rate for patients with AML (18). Kaplan–Meier analysis revealed that elevated NTMT1 expression was associated with shorter OS, PFI, and DSS in OSCC patients. However, multivariate Cox regression analysis indicated that NTMT1 expression was not an independent prognostic factor for overall survival. These results suggest that NTMT1 may not independently affect patient survival through a direct mechanism. Instead, its correlation with clinical prognosis may be mediated by its association with other clinicopathological covariates and the modulation of tumor biological behaviors—an observation consistent with our earlier finding that NTMT1 expression is closely correlated with aggressive clinicopathological features of OSCC. It should also be noted that bioinformatic analyses are inherently prone to certain biases due to the heterogeneity of public datasets and inherent limitations of computational algorithms, which may also contribute to the lack of independent prognostic value for NTMT1 identified in this study. Additionally, our functional analysis demonstrated that NTMT1 can promote cell cycle progression and impair intercellular tight junctions, implying that it acts as a progression-associated biological marker to accelerate the proliferation and metastatic potential of OSCC cells.

While our findings confirm that NTMT1 expression correlates with OSCC patient prognosis, the lack of independent prognostic significance in multivariate analysis prompts further exploration of its potential regulatory mechanisms—particularly its association with m6A methylation, a key epigenetic modification involved in OSCC progression (21). According to a study in head and neck squamous cell carcinoma, m6A modification mediates the epigenetic upregulation of NTMT1 in an M6A-dependent manner via the methyltransferase METTL3 (22). This study found that NTMT1 in OSCC had a relatively high positive correlation with HNRNPC and RBMX. Zhu et al. demonstrated that HNRNPC could promote the invasion and migration of OSCC cells by regulating abnormal glycolysis and mitochondrial respiration (23). Although the mechanism underlying the role of RBMX in OSCC remains unclear, studies have confirmed that it serves as a risk factor for the prognosis of OSCC patients (24, 25). These results suggest that NTMT1 may influence OSCC progression via HNRNPC and RBMX, or alternatively, that HNRNPC and RBMX may regulate the epigenetics of NTMT1—though the specific mechanism requires further investigation.

Tumor-infiltrating lymphocytes play a pivotal role in the OSCC progression. Specifically, the type, density, and spatial distribution of TILs are closely associated with the immunosuppressive status of the tumor microenvironment (26). In this study, multiple approaches were used to investigate the relationship between NTMT1 expression and immune cells in OSCC tissues. We found that NTMT1 expression was negatively correlated with the infiltration levels of various immune cell types, among which the correlation between NTMT1 and mast cells was the strongest. Mast cells play a complex and multifaceted role in OSCC. A study showed that mast cell counts are higher in OSCC and oral potentially malignant disorders (OPMDs) than in normal tissues, but decrease as OPMDs progress to OSCC, suggesting mast cells may exert a pro-inflammatory role in early oral carcinogenesis and potentially shift to a protective role in advanced stages (27). High mast cell density correlates with longer overall survival in OSCC, suggesting mast cells may exert a protective function, for instance by modulating immune responses to suppress tumor invasion (28). Whether NTMT1 promotes OSCC progression by reducing mast cell infiltration remains to be further verified. In addition, although NTMT1 expression shows a significant association with some other immune cell types, the correlation strength is relatively weak. Whether this implies that NTMT1 has a minor impact on tumor-infiltrating immune cells requires further investigation.

The direct regulatory effect of NTMT1 on tumor lethality also has been reported. Silencing NTMT1 can enhance the sensitivity of retinoblastoma cells to cisplatin (16). NTMT1 promotes the proliferation of npl4-regulated bladder cancer cells by upregulating cyclin D1 expression (17). Overexpression of NTMT1 can facilitate the proliferation and migration of AML cells (18). Specific degradation of NTMT1 can inhibit the proliferation of colorectal cancer cells by inducing G0/G1 cell cycle arrest (29). Finally, we overexpressed NTMT1 in OSCC cells to explore the influence of NTMT1 on malignant biological behavior of OSCC cells. The results confirmed that NTMT1 overexpression promoted the proliferation, migration and invasion of OSCC cells. Prior studies have established that NTMT1 is involved in regulating critical malignant phenotypes across multiple cancer types, and our work extends this understanding by confirming its capacity to drive the proliferation, migration, and invasion of OSCC cells. This alignment between our findings and existing literature not only reinforces the credibility of NTMT1’s pro-tumor function but also underscores the need to investigate its downstream signaling pathways in OSCC, thereby identifying novel intervention strategies.

This study confirmed that high NTMT1 expression is associated with the clinicopathological features and poor prognosis of OSCC patients, but failed to identify it as an independent prognostic factor. Thus, the prognostic significance of NTMT1 may partially stem from its correlation with other clinical features. Additionally, the impact of NTMT1 on OSCC progression may also be mediated through m6A methylation and tumor-infiltrating immune cells. Collectively, NTMT1 shows potential as a component of a multi-gene signature for OSCC, providing a new perspective for optimizing the diagnostic and therapeutic strategies for OSCC patients.

## Data Availability

The original contributions presented in the study are included in the article/**Supplementary Material**. Further inquiries can be directed to the corresponding authors.

## References

[1] Adam H Nishant A Zhen G . Lip and oral cavity squamous cell carcinoma. Hematol Oncol Clin North Am. (2021) 35:895–911. doi: 10.1016/j.hoc.2021.05.003, PMID: 34274176

[2] Gonçalves FA Bittencourt LDS Barbosa S Diel LF Bernardi L Matte C . Energy metabolic profile in oral potentially Malignant disorders and oral squamous cell carcinoma: A preliminary landscape of warburg effect in oral cancer. Mol Carcinog. (2025) 64:126–37. doi: 10.1002/mc.23831, PMID: 39412414

[3] Ali AN Ghoneim SM Ahmed ER Salam LOEA Saleh SMA . Cadherin switching in oral squamous cell carcinoma: A clinicopathological study. J Oral Biol Craniofac Res. (2023) 13:486–94. doi: 10.1016/j.jobcr.2023.05.001, PMID: 37293580 PMC10245331

[4] Nguyen TTH Sodnom-Ish B Choi SW Jung HI Cho J Kim SM . Salivary biomarkers in oral squamous cell carcinoma. J Korean Assoc Oral Maxillofac Surg. (2020) 46:301–12. doi: 10.5125/jkaoms.2020.46.5.301, PMID: 33122454 PMC7609938

[5] Hussain F Nair A Tharakan A . MicroRNA-based markers of oral tongue squamous cell carcinoma and buccal squamous cell carcinoma. Cureus. (2025) 17:e79733. doi: 10.7759/cureus.79733, PMID: 40161045 PMC11953619

[6] Meng Y Huang R . Site-specific methylation on α-N-terminus of peptides through chemical and enzymatic methods. Methods Enzymol. (2023) 684:113–33. doi: 10.1016/bs.mie.2023.02.008, PMID: 37230586 PMC10525076

[7] Meng Y Li Z He M Zhang Q Deng Y Wang Y . Characterizations of protein arginine deiminase 1 as a substrate of NTMT1: implications of Nα-methylation in protein stability and interaction. J Proteome Res. (2024) 23:4589–600. doi: 10.1021/acs.jproteome.4c00484, PMID: 39287128 PMC11452276

[8] Dong G Iyamu ID Vilseck JZ Chen D Huang R . Improved cell-potent and selective peptidomimetic inhibitors of protein N-terminal methyltransferase 1. Molecules. (2022) 27:1381. doi: 10.3390/molecules27041381, PMID: 35209173 PMC8874984

[9] Wang Z He Z Lin R Feng Z Li X Sui X . Evaluation of a plasma cell-free DNA methylation test for colorectal cancer diagnosis: a multicenter clinical study. BMC Med. (2024) 22:1–10. doi: 10.1186/s12916-024-03662-y, PMID: 39379942 PMC11462859

[10] Zhang J Song H Chen C Chen L Dai Y Sun P . Methyltransferase-like protein 11 A promotes migration of cervical cancer cells via up-regulating ELK3. Pharmacol Res. (2021) 172:105814. doi: 10.1016/j.phrs.2021.105814, PMID: 34450313

[11] Bindea G Mlecnik B Tosolini M Kirilovsky A Waldner M Obenauf AC . Spatiotemporal dynamics of intratumoral immune cells reveal the immune landscape in human cancer. Immunity. (2013) 39:782–95. doi: 10.1016/j.immuni.2013.10.003, PMID: 24138885

[12] Kalló G Bertalan PM Márton I Kiss C Csősz É . Salivary chemical barrier proteins in oral squamous cell carcinoma-alterations in the defense mechanism of the oral cavity. Int J Mol Sci. (2023) 24:13657. doi: 10.3390/ijms241713657, PMID: 37686462 PMC10487546

[13] Li Z Sun S Wang Y Hua Y Liu M Zhou Y . PA28γ coordinates the cross-talk between cancer-associated fibroblasts and tumor cells to promote OSCC progression via HDAC1/E2F3/IGF2 signaling. Cancer Lett. (2024) 594:216962. doi: 10.1016/j.canlet.2024.216962, PMID: 38768680

[14] Dong C Mao Y Tempel W Qin S Li L Loppnau P . Structural basis for substrate recognition by the human N-terminal methyltransferase 1. Genes Dev. (2015) 29:2343–8. doi: 10.1101/gad.270611.115, PMID: 26543161 PMC4691889

[15] Chen D Dong G Noinaj N Huang R . Discovery of bisubstrate inhibitors for protein N-terminal methyltransferase 1. J Med Chem. (2019) 62:3773–9. doi: 10.1021/acs.jmedchem.9b00206, PMID: 30883119 PMC6760264

[16] Li Z Zhang L Liu D Yang Z Xuan D Zhang Y . Knockdown of NRMT enhances sensitivity of retinoblastoma cells to cisplatin through upregulation of the CENPA/Myc/Bcl2 axis. Cell Death Discov. (2022) 8:14. doi: 10.1038/s41420-021-00622-w, PMID: 35013138 PMC8748520

[17] Lu BS Liu KL Yin YW Zhang YP Qi JC Zhao CM . A novel feedback regulation loop of METTL11A–MAFG–NPL4 promotes bladder cancer cell proliferation and tumor progression. FASEB J. (2025) 39:e70466. doi: 10.1096/fj.202402830R, PMID: 40171788

[18] Li J Zhang J Zhang P . METTL11A serves as a novel therapeutic target for acute myeloid leukemia through regulation of the p38-MAPK pathway. Transl Cancer Res. (2025) 14:4561–73. doi: 10.21037/tcr-2025-602, PMID: 40950680 PMC12432591

[19] Aguzzi A Maggioni D Nicolini G Tredici G Gaini RM Garavello W . MAP kinase modulation in squamous cell carcinoma of the oral cavity. Anticancer Res. (2009) 29:303–8. 19331166

[20] Brands MT Campschroer G Merkx MAW Verbeek ALM Dijk BAC Geurts SME . Second primary tumours after squamous cell carcinoma of the oral cavity. Eur J Surg Oncol. (2009) 47:1934–9. doi: 10.1016/j.ejso.2021.03.242, PMID: 33896667

[21] Huang J Li H Yang Z Liu R Li Y Hu Y . SALL4 promotes cancer stem-like cell phenotype and radioresistance in oral squamous cell carcinomas via methyltransferase-like 3-mediated m6A modification. Cell Death Dis. (2024) 15:139. doi: 10.1038/s41419-024-06533-9, PMID: 38355684 PMC10866932

[22] Zhao C Yu M Li Y . Pan-cancer analysis reveals the pro-oncogenic role of N6-methyladenosine (m6A)-regulated NTMT1 in head and neck squamous cell carcinoma. J Biochem Mol Toxicol. (2024) 38:e23603. doi: 10.1002/jbt.23603, PMID: 38014887

[23] Zhu W Wang J Liu X Xu Y Zhai R Zhang J . lncRNA CYTOR promotes aberrant glycolysis and mitochondrial respiration via HNRNPC-mediated ZEB1 stabilization in oral squamous cell carcinoma. Cell Death Dis. (2022) 13:703. doi: 10.1038/s41419-022-05157-1, PMID: 35963855 PMC9376070

[24] Jihua G Xiaole W Jun J Rong J . Underexpression of SRSF3 and its target gene RBMX predicts good prognosis in patients with head and neck cancer. J Oral Sci. (2020) 62:175–9. doi: 10.2334/josnusd.18-0485, PMID: 32132325

[25] Liu Y Nie J Huang Y Yang Y Su W Zhang Y . m6A-related genes ALKBH5 and RBMX as prognostic and progression biomarkers in Chinese oral squamous cell carcinoma patients. Arch Oral Biol. (2025) 170:106149. doi: 10.1016/j.archoralbio.2024.106149, PMID: 39643954

[26] Quan H Shan Z Liu Z Liu S Yang L Fang X . The repertoire of tumor-infiltrating lymphocytes within the microenvironment of oral squamous cell carcinoma reveals immune dysfunction. Cancer Immunol Immunother. (2020) 69:465–76. doi: 10.1007/s00262-020-02479-x, PMID: 31950224 PMC11027813

[27] Shrestha A Keshwar S Raut T . Evaluation of mast cells in oral potentially Malignant disorders and oral squamous cell carcinoma. Int J Dent. (2021) 2021:5609563. doi: 10.1155/2021/5609563, PMID: 34490052 PMC8418547

[28] Brockmeyer P Kling A Schulz X Perske C Schliephake H Hemmerlein B . High mast cell density indicates a longer overall survival in oral squamous cell carcinoma. Sci Rep. (2017) 7:14677. doi: 10.1038/s41598-017-15406-5, PMID: 29116177 PMC5677084

[29] Zhou Q Wu W Jia K Qi G Sun XS Li P . Design and characterization of PROTAC degraders specific to protein N-terminal methyltransferase 1. Eur J Med Chem. (2022) 244:114830. doi: 10.1016/j.ejmech.2022.114830, PMID: 36228414 PMC10520980

