# Supplementary material for: Prognostic significance of NTMT1 and its association with tumor progression in oral squamous cell carcinoma
Source: Front Oncol. 2026 Mar 11;16:1730415. doi: 10.3389/fonc.2026.1730415 (PMC13012952; doi:10.3389/fonc.2026.1730415)
Supplement: Supplementary file 1 [file DataSheet1.docx]

**Experimental Methods**

**Cell Culture**

Human oral squamous cell carcinoma cell lines SCC15 and CAL27 used in this study were purchased from the Cell Bank of the Chinese Academy of Sciences. Cells were cultured in DMEM medium (#12430054, Gibco, USA) supplemented with 10% fetal bovine serum (#10099133, Gibco), 100 U/mL penicillin, and 100 μg/mL streptomycin, and maintained in a humidified incubator at 37°C with 5% CO₂.

**Plasmid Transfection**

The NTMT1 overexpression plasmid and empty vector plasmid were constructed by Wuhan Miaoling Biotechnology Co., Ltd. Transfection was performed using Lipofectamine 300 (#L3000001, Invitrogen, USA). One day before transfection, cells were seeded into 6-well plates at a density of 5×10⁵ cells/well. For transfection, plasmid-P3000-transfection reagent complexes were prepared, incubated at room temperature for 20 minutes, and then added to the wells. The medium was replaced after 6 hours of culture, and transfection efficiency was verified by Western Blot at 48 hours post-transfection.

**Western Blot**

Cells were collected and lysed on ice using RIPA lysis buffer containing protease inhibitors, and the supernatant was obtained by centrifugation. After protein quantification using the BCA method, proteins were mixed with loading buffer and denatured. Proteins were separated by 10%-12% SDS-PAGE electrophoresis, then transferred onto PVDF membranes via wet transfer. The membranes were blocked with 5% non-fat milk for 1.5-2 hours, incubated with primary antibodies overnight at 4°C, and then with secondary antibodies for 1 hour at room temperature. After ECL chemiluminescence detection, ImageJ software was used to quantify the gray value of protein bands. GAPDH was used as the internal reference to calculate the relative expression level of the target protein.

**CCK8 Proliferation Assay**

Logarithmic phase cells were digested and adjusted to a density of 5×10³ cells/well, then seeded into 96-well plates with 100 μL cell suspension per well. Cells were cultured for 0 h, 24 h, 48 h, and 72 h respectively. At each time point, 10 μL of CCK8 reagent was added to each well, followed by incubation for 2 hours. The optical density (OD) value at 450 nm was measured using a microplate reader.

**Cell Colony Formation Assay**

Cells were digested to prepare a single-cell suspension and seeded into 6-well plates at a density of 500 cells/well. Cells were cultured in a 37°C incubator with 5% CO₂ for 10 days until visible colonies formed. Colonies were fixed with 4% paraformaldehyde and stained with 0.1% crystal violet. The number of colonies was counted, and the colony formation rate was calculated using the formula: Colony formation rate (%) = (Number of colonies / Number of seeded cells) × 100%.

**Cell Scratch Assay**

Cells were seeded into 6-well plates at a density of 2×10⁶ cells/well. When cell confluency reached 100%, a uniform scratch was made across the cell monolayer using a 100 μL pipette tip. Cell debris was removed by washing with PBS, and the cells were cultured in serum-free medium. Images of the scratch were captured at 0 h and 24 h, and ImageJ software was used to measure the scratch width and calculate the scratch healing distance.

**Transwell Migration Assay**

The upper chamber of a Transwell insert (pore size: 8 μm) was hydrated with serum-free medium for 30 minutes. Cells were adjusted to a density of 5×10⁴ cells/100 μL, and 100 μL of the cell suspension was added to the upper chamber. Meanwhile, 600 μL of medium containing 10% FBS was added to the lower chamber. After 24 hours of culture, non-migrated cells in the upper chamber were wiped off, and migrated cells were fixed and stained. The number of migrated cells was counted in 5 randomly selected fields under a 200× microscope, and the average value was calculated.

**Transwell Invasion Assay**

Matrigel matrix was diluted at a ratio of 1:8 and coated onto the upper chamber of a Transwell insert, which was then incubated at 37°C for 6 hours to allow gelation. Cells were adjusted to a density of 1×10⁵ cells/100 μL, and 100 μL of the cell suspension was added to the upper chamber, while medium containing FBS was added to the lower chamber. After 48 hours of culture, the subsequent steps of fixation, staining, and cell counting were performed as described in the Transwell migration assay, and the number of invasive cells was statistically analyzed.
